# Cirrus: An Automated Mammography-Based Measure of Breast Cancer Risk Based on Textural Features

**DOI:** 10.1093/jncics/pky057

**Published:** 2018-12-07

**Authors:** Daniel F Schmidt, Enes Makalic, Benjamin Goudey, Gillian S Dite, Jennifer Stone, Tuong L Nguyen, James G Dowty, Laura Baglietto, Melissa C Southey, Gertraud Maskarinec, Graham G Giles, John L Hopper

**Affiliations:** 1 Centre for Epidemiology and Biostatistics, Melbourne School of Population and Global Health, The University of Melbourne, Parkville, Victoria, Australia; 2 Faculty of Information Technology, Monash University, Clayton, Victoria, Australia; 3 IBM Australia - Research, Southbank, Victoria, Australia; 4 Curtin UWA Centre for Genetic Origins of Health and Disease, Curtin University, and the University of Western Australia, Perth, Western Australia, Australia; 5 Department of Clinical and Experimental Medicine, University of Pisa, Pisa, Italy; 6 Department of Pathology, University of Melbourne, Carlton, Victoria, Australia; 7 Precision Medicine, School of Clinical Sciences at Monash Health, Monash University, Clayton, Victoria, Australia; 8 University of Hawaii Cancer Center, Honolulu, HI; 9 Cancer Epidemiology Centre, Cancer Council Victoria, Melbourne, Victoria, Australia

## Abstract

**Background:**

We applied machine learning to find a novel breast cancer predictor based on information in a mammogram.

**Methods:**

Using image-processing techniques, we automatically processed 46 158 analog mammograms for 1345 cases and 4235 controls from a cohort and case–control study of Australian women, and a cohort study of Japanese American women, extracting 20 textural features not based on pixel brightness threshold. We used Bayesian lasso regression to create individual- and mammogram-specific measures of breast cancer risk, Cirrus. We trained and tested measures across studies. We fitted Cirrus with conventional mammographic density measures using logistic regression, and computed odds ratios (OR) per standard deviation adjusted for age and body mass index.

**Results:**

Combining studies, almost all textural features were associated with case–control status. The ORs for Cirrus measures trained on one study and tested on another study ranged from 1.56 to 1.78 (all *P *<* *10^−6^). For the Cirrus measure derived from combining studies, the OR was 1.90 (95% confidence interval [CI] = 1.73 to 2.09), equivalent to a fourfold interquartile risk ratio, and was little attenuated after adjusting for conventional measures. In contrast, the OR for the conventional measure was 1.34 (95% CI = 1.25 to 1.43), and after adjusting for Cirrus it became 1.16 (95% CI = 1.08 to 1.24; *P *=* *4 × 10^−5^).

**Conclusions:**

A fully automated personal risk measure created from combining textural image features performs better at predicting breast cancer risk than conventional mammographic density risk measures, capturing half the risk-predicting ability of the latter measures. In terms of differentiating affected and unaffected women on a population basis, Cirrus could be one of the strongest known risk factors for breast cancer.

It is well established that there is information in a mammogram that predicts a woman’s risk of a future breast cancer. Mammographic density has conventionally been defined as the white or bright regions on a mammographic image. Considerable research has shown that, after adjusting for age and body mass index (BMI), the residuals of the absolute and percentage values of conventional mammographic density are highly correlated with one another, and both sets of residuals have been found by many studies to be associated with breast cancer risk (1). Residuals are the appropriate way to consider mammographic density as a risk factor for breast cancer because, across the age range relevant to most mammographic density studies, age and BMI are negatively associated with conventional mammographic density measures (2) but positively associated with breast cancer risk. These residuals are also highly correlated over time (3,4). 

For the conventional measures of mammographic density, once adjusted for age and BMI, the risk increases by about 1.4-fold per adjusted standard deviation (3,5,6), equivalent to an approximately twofold interquartile risk ratio (IQRR) (7). In comparison, the risk gradient for the current best polygenic breast cancer risk score based on common variants (single-nucleotide polymorphisms; SNPs), is about 1.6-fold per standard deviation or a threefold IQRR (8,9).

The bench mark for measuring mammographic density has been a computer-assisted thresholding technique using Cumulus software (Sunnybrook Health Sciences Centre, Toronto, Canada) (10). A digital version of the mammographic image is divided into two segments based on a pixel brightness threshold chosen by the measurer; one segment represents what the measurer considers to be the white or bright regions (ie, the dense tissues) and the remainder of the breast is considered to be nondense.

Although highly repeatable across trained measurers, the semiautomated Cumulus measurements involve subjective judgment and are too labor intensive for clinical use. Automated measures of conventionally defined mammographic density, such as AutoDensity (11), LIBRA (12), and Volpara (13) (which aims to estimate the volume of dense tissue), have been developed. We have also found, using Cumulus software, but in effect defining mammographic density at higher pixel brightness thresholds, that stronger risk gradients can be obtained (5,6,14). This raises the possibility that better risk-predicting measures could be found by considering characteristics of a mammogram other than the conventional concept of mammographic density.

All the risk-predicting measures above summarize a mammographic image by a single quantity: an estimate of the area or volume of dense tissue. This is equivalent to counting the number of pixels above a brightness threshold, and uses first-order statistical information only. Even the volumetric Volpara measure reduces to the same process, in that it defines mammographic density as the number of voxels (3 D pixels) deemed to be dense.

In this article, we describe an alternative and agnostic approach to discovering risk-predicting information in a mammographic image. We used machine learning to capture the combination of textural and spatial information, not necessarily observable by a human, that best predicts breast cancer risk. In contrast to conventional approaches, we used second-order statistical information (ie, measures of variability and interrelations between pixels).

We tested the risk predicting capability of each measure, trained on a specific study, on the other studies. We then combined these features and studies to produce a fully automated mammography-based risk measure that we named Cirrus. Although we are not the first to take such an approach [see Gastounioti et al. (15) for a summary of texture-based approaches], we consider that our large sample sizes, the validation across populations and designs, and the way that we handle the highly correlated textural features are strengths.

## Methods and Materials

### Subjects

We analyzed digitized film mammograms of women with breast cancer (cases) and women without a diagnosis of breast cancer (controls) from three studies:

1. Caucasian cohort (Australia): a nested case−control study of 669 cases and 2629 controls from the Melbourne Collaborative Cohort Study for whom we obtained 36 897 mammograms (average six visits per woman) (16).
2. Caucasian case−control (Australia): a case−control study of 384 case patients with invasive disease and 1314 control subjects from the Australian Breast Cancer Family Study and the Australian Mammographic Density Twins and Sisters Study for whom we obtained 6243 mammograms (average two visits per woman) (5).
3. Japanese cohort (Hawaii): a nested case−control study within the Multiethnic Cohort Study of Japanese American women living in Hawaii, USA, consisting of 292 cases (23% ductal carcinoma in situ and 77% invasive) and 292 controls, for whom we obtained 3018 mammograms (average three visits per woman) (17).

Supplementary Table 1 (available online) summarizes key characteristics of the studies and subjects. All participants gave written informed consent and the studies were approved by appropriate human research ethics committees [see (5),(16), and (17), respectively].

All film mammograms were digitizsed. Because our software automatically handled removal of pectoral muscles from craniocaudal (CC) views, but not from mediolateral oblique (MLO) mammograms, analysis was restricted to CC view mammograms. (Our pilot study of around 600 MLO views found that the risk prediction was no different between CC and MLO views.) We used both left and right mammograms at the same visits, and multiple vists when available. The median number of mammograms per woman was 10 (Australian cohort study), 2 (Australian case–control study), and 5 (Japanese American study).

### Quality Control

All datasets underwent quality control to remove inappropriate mammograms (eg, negative images, MLOs misclassified as CCs, damaged film mammograms, and case patient mammograms used for their diagnosis) as well as mammograms that failed the automatic preprocessing stage by being incorrectly segmented (eg, when the range of contrast was abnormally low). After quality control, less than 3% of mammograms in any dataset were removed.

### Image Analysis

We developed and applied an automatic preprocessing algorithm that uses image-processing techniques to segment the breast from the background noise, and to remove artefacts and labels before feature extraction (Supplementary Methods, available online).

### Feature Extraction

We applied algorithms to extract features of potential interest. We required features to be *invariant* to rotation and translation, so that a mammogram will yield the same features irrespective of the positioning and orientation of the breast. In the image-processing literature, texture refers to the relationship between pixels in a neighborhood. Bringing second-order information, texture essentially provides information on the types of patterns present in an image; eg, whether areas of the image are smooth or rough, or whether the rough and smooth areas are scattered across an image or clustered together, etc.

We chose the gray-level co-occurrence matrix (GLCM) class of features (18–20), based on the statistical properties of neighboring pixels. Many GLCM textures act as analogues of quantities found in the physical sciences. For example, homogeneity measures the degree of “scatteredness” of the texture within an image, so images with large areas of similar intensity pixels have a higher degree of homogeneity than those composed of a large number of small dissimilar regions.

A total of 20 GLCM features common in the literature were extracted from all mammograms (see Table 1). Importantly, because the characteristics and behavior of digitizers vary by manufacturer and study, we modified the standard GLCM feature algorithm so that our features would be resistant to digitizer settings. All image analysis was performed using the MATLAB computing platform. More details on the GLCM feature extraction can be found in the Supplementary Methods (available online).

**Table 1.** Marginal odds ratio (OR) per unadjusted standard deviation, with 95% confidence interval (CI) and *P* value, for each feature from each study

Feature	Caucasian cohort (Australia)		Caucasian case–control (Australia)		Japanese cohort (Hawaii)	
	OR (95% CI)	*P*	OR (95% CI)	*P*	OR (95% CI)	*P*
Autocorrelation	0.89 (0.82 to 0.96)	.004	1.14 (1.01 to 1.29)	.03	1.18 (0.98 to 1.43)	.07
Cluster prominence	1.11 (1.02 to 1.20)	.01	1.05 (0.93 to 1.19)	.4	0.92 (0.77 to 1.11)	.4
Cluster shade	1.13 (1.04 to 1.22)	.003	0.94 (0.83 to 1.07)	.3	0.87 (0.73 to 1.05)	.1
Contrast	0.57 (0.52 to 0.63)	10^−28^	0.60 (0.50 to 0.72)	3 × 10^−8^	0.66 (0.53 to 0.81)	6 × 10^−5^
Correlation	1.75 (1.59 to 1.94)	10^−28^	1.68 (1.40 to 2.01)	2 × 10^−8^	1.56 (1.26 to 1.92)	4 × 10^−5^
Difference entropy	0.58 (0.54 to 0.64)	10^−31^	0.60 (0.52 to 0.70)	10^−11^	0.67 (0.55 to 0.81)	4 × 10^−5^
Difference variance	0.57 (0.52 to 0.63)	10^−28^	0.60 (0.50 to 0.72)	3 × 10^−8^	0.66 (0.53 to 0.81)	6 × 10^−5^
Dissimilarity	0.58 (0.53 to 0.64)	10^−29^	0.61 (0.52 to 0.71)	10^−10^	0.66 (0.54 to 0.81)	4 × 10^−5^
Energy	1.18 (1.09 to 1.27)	4 × 10^−5^	1.01 (0.90 to 1.15)	.8	0.90 (0.75, 1.08)	.3
Entropy	0.69 (0.63 to 0.75)	10^−18^	0.72 (0.63 to 0.82)	9 × 10^−7^	0.82 (0.68 to 0.99)	.04
Homogeneity	1.71 (1.56 to 1.88)	10^−30^	1.64 (1.41 to 1.90)	10^−10^	1.51 (1.24 to 1.84)	4 × 10^−5^
Information correlation 1	0.58 (0.53 to 0.63)	10^−32^	0.58 (0.51 to 0.67)	10^−13^	0.63 (0.52 to 0.77)	4 × 10^−6^
Information correlation 2	1.80 (1.64 to 1.98)	10^−33^	1.86 (1.58 to 2.19)	10^−13^	1.80 (1.47 to 2.20)	1 × 10^−8^
Maximum probability	1.09 (1.00 to 1.17)	.04	0.91 (0.81 to 1.03)	.2	0.84 (0.70 to 1.01)	.06
Moment normalized inverse difference	1.75 (1.58 to 1.93)	10^−28^	1.67 (1.40 to 1.99)	1 × 10^−8^	1.53 (1.24 to 1.87)	5 × 10^−5^
Normalized inverse difference	1.72 (1.56 to 1.89)	10^−29^	1.64 (1.41 to 1.92)	10^−10^	1.51 (1.24 to 1.84)	4 × 10^−5^
Sum average	0.89 (0.82 to 0.97)	.005	1.11 (0.99 to 1.26)	.08	1.18 (0.98 to 1.41)	.09
Sum variance	0.94 (0.87 to 1.01)	.1	1.28 (1.14 to 1.45)	6 × 10^−5^	1.28 (1.06 to 1.54)	.009
Sum entropy	0.75 (0.69 to 0.81)	10^−12^	0.83 (0.73 to 0.93)	.002	0.93 (0.77 to 1.11)	.4
Variance	0.88 (0.81 to 0.95)	.002	1.12 (0.99 to 1.27)	.07	1.17 (0.97 to 1.41)	.1

### Statistical Analysis

For each feature, the information for a given woman was taken to be the median of her features across all her mammograms because this increased the amount of information for risk prediction and produces a more stable predictor. Marginal (one-at-a-time) estimation of the association between the 20 GLCM features and breast cancer risk, unadjusted for covariates, was first performed using logistic regression and presented as the odds ratio (OR) per standard deviation of the unadjusted cross-sectional measure.

A risk measure, Cirrus, was computed from features by applying the Bayesian lasso regression procedure (21), which is based on a Bayesian interpretation of the lasso (22) penalty that automatically estimates the regularization parameter to avoid statistical instability due to collinearity and makes best use of all the available features [see (23)].The logistic regression models were estimated by drawing 10 000 samples from the posterior distribution, with the first 1000 discarded as burn-in samples, and using the Bayesreg Bayesian regression software (24) in MATLAB. The measure of breast cancer risk was a linear combination of the estimated coefficients and the image features.

A separate Cirrus measure was constructed from each dataset. Each trained measure was tested on the given dataset, to assess its maximum risk prediction, and then tested on each of the other two independent datasets, to see the extent to which the discovery process could be externally validated.

We assessed risk-predicting performance using logistic regression and adjusting for age and BMI. Risk gradients were presented as the change in the age- and BMI-adjusted OR per unit change in the standard deviation of the residual of the measure after adjusting for age and BMI using the controls, following the OPERA concept (7). The nominal *P* values were determined using the Wald test. As shown in the Supplementary Methods (available online), for a continuous risk factor satisfying a normality assumption and a relatively rare disease, IQRR = $\Phi \left(log(OR)-b\right)/\Phi \left(a-log(OR)\right)$, where Φ is the cumulative distribution function of the standard normal distribution and $a\approx \Phi^{-1}\left(0.25\right)=-0.6745$ and $b\approx \Phi^{-1}\left(0.75\right)=0.6745$. The relationship between log (OR) and the area under the receiver operator curve (AUC) are also shown in the Supplementary Methods (available online).

We also created a Cirrus measure trained on the combined data, and fitted it to the combined data using logistic regression adjusting for age and BMI, with and without the conventional risk measures of absolute and percentage mammographic density power transformed and adjusted for age and BMI. Based on the Box–Cox transformation, we used the fourth root of absolute density and the cube root of percentage density. We used the conventional mammographic density measures, created using the semiautomated computer software Cumulus (10), from the published cohort (16) and case−control (5,17) studies, and the subjects for whom BMI data were available.

## Results

Table 1 shows that, for 11 of the 20 GLCM image features (contrast, correlation, dissimilarity, homogeneity, difference variance, difference entropy, entropy, information correlation 1, information correlation 2, normalized inverse difference, and moment normalized inverse difference), the directions and magnitudes of their risk associations were similar across all studies. This indicates that there is a relationship of features to breast cancer risk that is robust to study variation.

Table 2 shows that the OR per adjusted standard deviation of the Cirrus measures trained on each study were highest when tested on that study itself, and in the range of 1.72 to 1.92. Most importantly, the cross-study replication associations were also high, ranging from 1.56 to 1.78 (all *P *<* *10^–^^6^). All the replication log(OR)s were within 20% of their in-sample training log(OR)s. The strongest cross-validation was for the Cirrus measure trained on the Australian cohort study and replicated on the study of Japanese American women living in Hawaii.

**Table 2.** Odds ratio per standard deviation after adjusting for age and BMI (95% confidence intervals) for the Cirrus measures trained on one dataset and tested on the same (diagonal) or another (off-diagonal) dataset

Training dataset	Testing dataset		
	Caucasian	Caucasian	Japanese American
	Cohort	case–control	cohort
	(Australia)	(Australia)	(Hawaii)
Caucasian	1.83 (1.65 to 2.03)	1.60 (1.41 to 1.82)	1.78 (1.46 to 2.17)
Cohort			
(Australia)			
Caucasian	1.56 (1.43 to 1.72)	1.72 (1.52 to 1.95)	1.75 (1.44 to 2.12)
Case–control			
(Australia)			
Japanese	1.58 (1.43 to 1.75)	1.61 (1.40 to 1.86)	1.92 (1.57 to 2.36)
cohort			
(Hawaii)			

**Figure 1.**
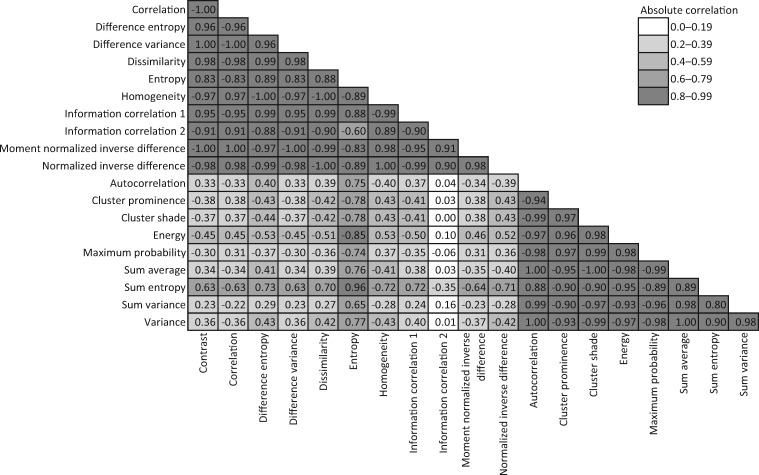
Correlations between the 20 textural features.

Table 3 shows that, when all three datasets were combined, nearly all the GLCM features were associated with case–control status. Figure 1 shows that the 11 GLCM features that were consistently associated across studies were highly correlated with each other (all *r *>* *0.9). These features also had similar absolute log(OR)s in Table 2. Figure 1 shows that the other nine GLCM features were also strongly correlated with each other, and Table 3 suggests that they had similar but lower absolute log(OR)s. Most pairs of features from the two different sets of 11 and 9 GLCM features were weakly correlated (absolute *r *<* *0.4). When we repeated the analyses using measures based on a single mammogram (the earliest) we found the general findings of Table 3 were repeatable across studies, although with greater variation across studies, justifying our use of the measures based on averaging over all mammograms.

**Table 3.** Marginal odds ratio (OR) per standard deviation after adjusting for age and body mass index, with 95% confidence interval (CI) and *P* value, for each feature from analysis of the combined dataset

Feature	OR (95% CI)	*P*
Autocorrelation	1.01 (0.94 to 1.08)	.9
Cluster prominence	1.06 (0.99 to 1.14)	.09
Cluster shade	1.03 (0.96 to 1.10)	.5
Contrast	0.55 (0.49 to 0.62)	10^−22^
Correlation	1.82 (1.62 to 2.05)	10^−23^
Difference entropy	0.57 (0.52 to 0.63)	10^−32^
Difference variance	0.55 (0.49 to 0.62)	10^−22^
Dissimilarity	0.57 (0.51 to 0.63)	10^−28^
Energy	1.08 (1.01 to 1.16)	.03
Entropy	0.70 (0.65 to 0.76)	10^−17^
Homogeneity	1.75 (1.59 to 1.93)	10^−29^
Information correlation 1	0.55 (0.50 to 0.60)	10^−35^
Information correlation 2	1.97 (1.77 to 2.20)	10^−33^
Maximum probability	0.99 (0.92 to 1.07)	.9
Moment normalized inverse difference	1.81 (1.61 to 2.03)	10^−23^
Normalized inverse difference	1.76 (1.59 to 1.94)	10^−28^
Sum average	1.00 (0.93 to 1.07)	1.0
Sum entropy	0.79 (0.74 to 0.85)	10^−9^
Sum variance	1.09 (1.01 to 1.17)	.02
Variance	0.99 (0.92 to 1.07)	.8

Table 4 shows the skewness and excess kurtosis of each of the 20 features in the combined dataset, along with the posterior standard deviation and standardized weight used to create the final Cirrus measure (see Supplementary Methods, available online, for more details). This Cirrus measure was independent of age and weakly negatively associated with BMI (*r* = −0.1).

**Table 4.** The skewness and excess kurtosis of each of the 20 features in the combined dataset, along with the posterior standard deviation (SD) and standardized weight used to create the final Cirrus risk measure

Feature	Skewness	Excess kurtosis	Posterior SD	Standardized weight
Autocorrelation	0.34	0.40	1.651	0.218
Cluster prominence	−0.26	0.33	0.008957	1.630
Cluster shade	−0.34	0.21	0.07228	0.267
Contrast	3.17	19.5	26.020	0.397
Correlation	−3.18	19.6	235.51	−0.321
Difference entropy	1.14	2.37	9.0306	−2.156
Difference variance	3.17	19.5	25.889	0.399
Dissimilarity	1.79	5.77	42.727	0.429
Energy	0.03	0.30	35.733	0.675
Entropy	0.60	0.63	17.761	−1.452
Homogeneity	−1.51	3.88	91.983	−0.983
Information correlation 1	1.09	2.03	35.328	−1.338
Information correlation 2	−2.17	8.14	136.29	−1.193
Maximum probability	−0.33	0.48	22.878	−1.473
Moment normalized inverse difference	−2.96	16.9	1976.2	1.096
Normalized inverse difference	−1.65	4.81	392.33	−0.600
Sum average	0.33	0.39	5.2236	−0.228
Sum entropy	0.19	0.002	19.135	1.919
Sum variance	0.26	0.46	0.6445	0.664
Variance	0.34	0.38	1.5106	−0.795

**Figure 2.**
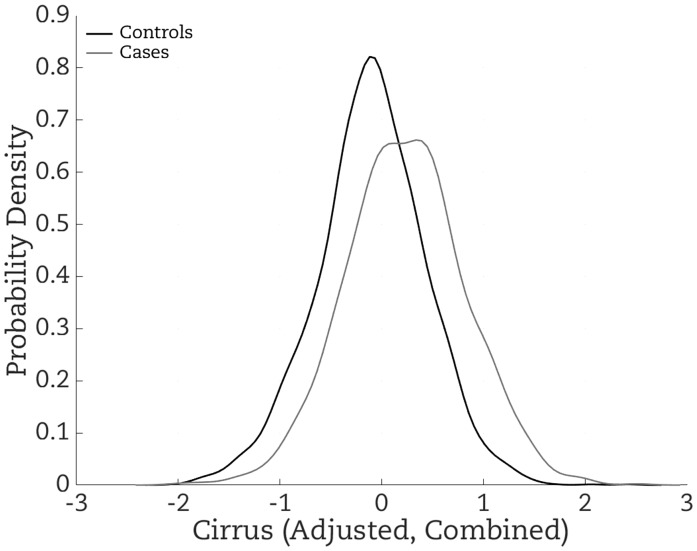
Distribution of the Cirrus measure created on the combined studies, adjusted for age and BMI, for cases (**gray line**) and controls (**dark line**). Risk increases with increasing Cirrus. The difference in the mean between cases and controls is equal to the log of the OR per standard deviation, which in turn is linearly related to the area under the receiver operator curve (AUC) in the range of 0.5 to 0.7; see theory and references in the Supplementary Methods (available online). See also Figure 3, which shows the corresponding receiver operator curve.

For the Cirrus risk measure (Cirrus adjusted for age and BMI) constructed from combining all datasets and all features and adjusted for age and BMI (see Figure 2), the OR per adjusted standard deviation was 1.90 (95% CI = 1.73 to 2.09) (Table 5), close to the value of 1.86 based on the standardized difference in means for case and controls shown in Figure 2 being 0.622 and the theory explained in the Supplementary Methods (available online). In comparison, the ORs per adjusted standard deviation for the risk factors based on absolute and percentage density measures were 1.34 and 1.38, respectively.

**Table 5.** Odds ratio (OR) adjusted for age and body mass index (BMI) for the mammography-based risk measures, age- and BMI-adjusted Cirrus, absolute and percentage mammographic density, and log BMI, fitted alone and in combination, from analysis of the combined dataset

Feature	OR (95% CI)	*P*
Cirrus	1.90 (1.73 to 2.09)	10^−38^
Absolute mammographic density	1.34 (1.25 to 1.43)	10^−17^
Percentage mammographic density	1.38 (1.29 to 1.48)	10^−20^
Log BMI	1.07 (0.99 to 1.15)	.07
Cirrus	1.76 (1.59 to 1.95)	10^−27^
Absolute mammographic density	1.16 (1.08 to 1.24)	4 × 10^−5^
Log BMI	1.04 (0.96 to 1.12)	.3
Cirrus	1.74 (1.57 to 1.93)	10^−25^
Percentage mammographic density	1.16 (1.07 to 1.25)	2 × 10^−4^
Log BMI	1.04 (0.97 to 1.12)	.3
Cirrus	1.74 (1.56 to 1.93)	10^−24^
Absolute mammographic density	1.11 (1.00 to 1.23)	.04
Percentage mammographic density	1.06 (0.95 to 1.19)	.3
Log BMI	1.04 (0.97 to 1.12)	.3

Table 5 also shows that the log(OR) for the Cirrus measure was reduced by less than 10% after adjusting for the conventional measures, and the predicted IQRR was about 4.2-fold (95% CI = 3.3 to 5.4).

In contrast, the OR for the conventional measure was 1.34 (95% CI = 1.25 to 1.43), and after adjusting for Cirrus it became 1.16 (95% CI = 1.08 to 1.24) (*P *=* *4 × 10^−^^5^). This was nearly a halving in log(OR), and a similar result applied to percentage density. The correlations between the risk estimates for Cirrus and the risk estimates for the absolute and percentage measures of conventional mammographic density were −0.3 and −0.4, respectively. When the two conventional mammographic density measures were modeled together, the percentage measure was not significant, consistent with risk being best captured by the absolute measure.

**Figure 3.**
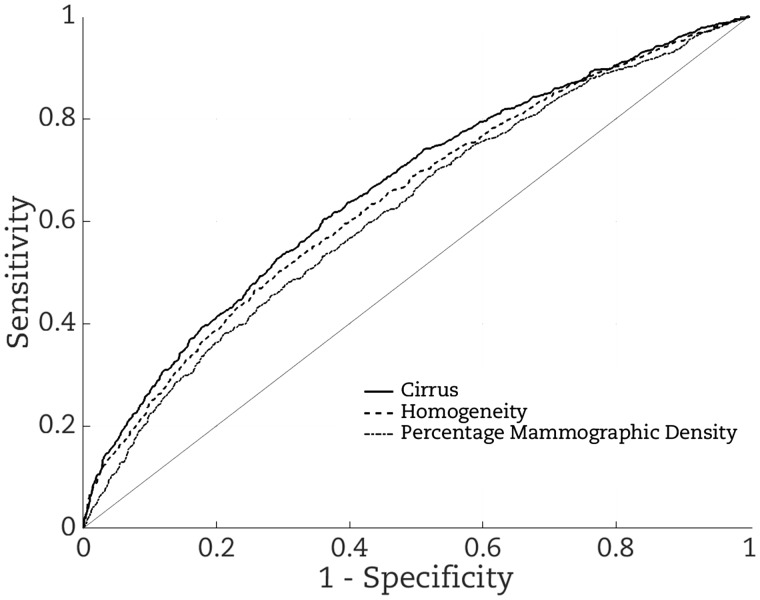
Receiver operator curves based on fitting: Cirrus created on the combined studies (**continuous line**); homogeneity alone (**dashed line**); and percentage mammographic density (**dotted line**). The corresponding areas under the receiver operator curves are 0.662, 0.642, and 0.620, respectively.

Figure 3 shows that, from considering the receiver operator curves, the Cirrus measure gives better risk discrimination (AUC = 0.662; 95% CI = 0.635 to 0.690) than does a Cirrus measure based on homogeneity alone (AUC = 0.642; 95% CI = 0.615 to 0.670), which gives better risk discrimination than does percentage mammographic density measure (AUC = 0.620; 95% CI = 0.593 to 0.648).

## Discussion

From applying machine learning to textural features of film mammograms, we have found novel information that predicts breast cancer risk. This finding was consistent and replicated across three studies using different designs and populations. Given replication and generalizability are key issues to achieve our aims, the similarities and differences in sample characteristics across these studies (details of which are summarized in Supplementary Table 1 [available online] and can be examined in detail by reference to the cited papers) has been advantageous. Analyses suggested that the textural features that differed in mean between the Caucasian and Japanese women were those that did not, of themselves, predict breast cancer risk (data not shown).

Our new mammography-based risk measure, Cirrus, is fully automated. It was better at predicting breast cancer risk than the conventional mammographic density risk measure and captured half of that measure’s risk-predicting ability. In terms of differentiating affected and unaffected women of the same age on a population basis, Cirrus is one of the strongest risk factors for breast cancer with an IQRR that could be as high as fourfold.

The risk discrimination of our Cirrus risk measures trained on a given dataset and tested on the other two were similar across all three datasets. The largest decrease in risk discrimination from training data to test data was only 20% on the log(OR) scale, and this was from training on the smallest sample (Japanese American women living in Hawaii) and testing on Australian women. The strong consistency across studies suggests that the identified risk-predicting features and their risk gradients are reliable predictors of breast cancer. It also suggests that our modification to the GLMC technique to make it resistant to regularity and collinearity effects across manufacturers and digitizers was successful.

An intriguing aspect of our findings is that the texture features used by the Cirrus measure are not based on absolute brightness thresholds, as are conventional mammographic density measures and the newer ones, *Altocumulus* and *Cirrocumulus*, defined at higher pixel brightness thresholds. Cirrus uses only *relative* brightness between pixels, yet achieves superior performance than the conventional measure across all studies, and has similar risk-predicting performance as do Altocumulus and Cirrocumulus for the Australian case–control study (5).

Our new study, and recent studies of Altocumulus and Cirrocumulus (5,6,14), raise the question as to whether the amount of what has conventionally been considered to be dense tissue should continue to be viewed as the gold standard of mammography-based risk estimation. The strong risk association we found with second-order textural information suggests that it may be a combination of the quantity and the spatial configuration of specific types of tissue that underlies the biological mechanisms determining breast cancer risk.

Almost all of the 20 GLCM features we studied were associated with breast cancer risk. Our statistical method helped us extract most of the information from the feature set even though they were correlated, and had some similarities to that used by Yaghian et al. (25) and Wang et al. (26) to find specific textural features predictive of masking and risk. There was a high correlation between some textural features, meaning that many capture the same textural information. This high level of collinearity likely explains the apparent lack of concordance in specific findings across studies using GLCM-type features [eg, Huo et al. (27),Wang et al. (26)]; when features are so highly correlated it is largely at random which features will be ranked as the “best” from analysis of any given dataset. Our aim was to build the best predictor, not to find the best individual predictor(s), so we used the Bayesian shrinkage procedure because it is known to be a better way to achieve our aim (23). This distinguishes our work from the previous studies (26,27).

For example, using a standard lasso procedure, Wang et al. (26) selected sum average, which tends to identify dispersed patterns of density, as the best predictor of risk. We, however, found no evidence that this feature was a major predictor of disease. They also noted that “the features that were not selected by lasso are not necessarily non-predictive of risk.” On the other hand, Wang et al. commented that “it was slightly surprising that … some previously reported texture features such as … contrast … were not selected, although contrast was significantly and negatively associated with risk in both the training and validation studies, in line with Huo et al.” We too found that contrast was negatively associated with risk, and this was highly significant in our study. A study of our risk predictors and statistical approaches with those of Wang et al. on the same dataset would be instructive.

Given our aim was to build the best predictor, use of the Bayesian lasso procedure mitigates the instability problem from procedures that try to first select features by estimating associations for all features. Notwithstanding, homogeneity was one of the strongest associated features, almost as good as the combination in predicting risk, and performed better than the conventional mammographic density measures (data not shown). However, considering that feature alone gave poorer internal and external risk prediction than using the combined measure, Cirrus, as is illustrated in Figure 3.

Supplementary Figure 1 (available online) shows mammograms from women with extreme (high and low) Cirrus risk measures, but whose conventional mammographic density risk measures were average (almost all between the 30th and 70th percentiles). The low-risk Cirrus images appear to be slightly darker overall.

Supplementary Figure 2 (available online) shows the same mammograms after processing and quantizing by the GLCM algorithm (see the Supplementary Methods, available online). The differences between the high- and low-risk Cirrus mammograms are clearer. The low-risk Cirrus mammograms have a scattered pattern with thin spiderwebs of brightness cutting through the darker regions. In contrast, the high-risk Cirrus mammograms appear to be composed of several large, well-defined, homogeneous connected regions. This would appear to reflect the homogeneity textural feature that we identified to be an important predictor of risk. Given its simple interpretation, homogeneity has the potential to be a useful new biomarker for breast cancer risk.

Some strengths of our study are our consistent findings related to textural features and strengths of association within and between study findings despite the variation in 1) ethnic origin of women, 2) the machines used to produce the mammograms, and 3) the digitization of mammograms. The strong cross-predictive performance suggests that we selected important aspects of a mammogram that are robust to differences in image acquisition. Cirrus appears to be essentially uncorrelated with age, and only weakly correlated with BMI and with both the absolute and percentage measures of conventional density. Further analyses (data not shown) suggest Cirrus is not highly correlated with family history and weakly corelated with number of live births and menopause status similar to Cumulus, and we are conducting detailed analyses of these issues as we did for Cumulus (2) for future publication. There appears to be room for improvement by, eg, extending the measure to incorporate more conventional mammographic risk features, such as dense area–like quantities defined by different pixel brightness thresholds (5,6).

The two main weaknesses of our study are 1) we combined interval and screen-detected cancers, which could weaken the ability of our measure to predict specifically risk or specifically masking and 2) our study was composed entirely of digitized film mammograms. Although the GLCM features account for some differences between images and machines, there is no guarantee that our current Cirrus measure will perform as well when ported to digital mammography; it could require further modification. It might also be that different features, or different weightings of features, provide better predictors of risk from digital mammograms. Not being able to readily identify and “see” the Cirrus measure and the individual features that underlie this phenomenon might be considered another weakness, and we have tried to address this. However, given that the consequence of applying machine learning is an automated mammography-based risk measure that does not require visual measurement, this might not be an impediment to future clinical use of measures like Cirrus when based on machine learning applied to digital mammograms.

In conclusion, we have used machine learning to create a new and fully automated mammography-based risk measure that has reliability within and across studies. Note that although we averaged mammograms to discover the predictor, we have created a risk predictor applicable to a single mammogram, thus allowing future studies of changes in time for a particular woman. One of the powerful features of using machine learning to create an automated measure of risk is that is it not necessary to have a human interpretation of the features—the human perception of the features will not be used on their own in practice. Our risk measure performs better at predicting breast cancer risk than the conventional mammographic density risk measures and captures half the risk-predicting ability of those measures. In terms of differentiating affected and unaffected women on a population basis, Cirrus could be one of the strongest known risk factors for breast cancer.

## Notes

Affiliations of authors: Centre for Epidemiology and Biostatistics, Melbourne School of Population and Global Health, The University of Melbourne, Parkville, Victoria, Australia (DFS, EM, BG, GSD, JS, TLN, JGD, GGG, JLH); Faculty of Information Technology, Monash University, Clayton, Victoria, Australia (DFS); IBM Australia - Research, Southbank, Victoria, Australia (BG); Curtin UWA Centre for Genetic Origins of Health and Disease, Curtin University, and the University of Western Australia, Perth, Western Australia, Australia (JS); Department of Clinical and Experimental Medicine, University of Pisa, Pisa, Italy (LB); Department of Pathology, University of Melbourne, Carlton, Victoria, Australia (MCS); Precision Medicine, School of Clinical Sciences at Monash Health, Monash University, Clayton, Victoria, Australia (MCS); University of Hawaii Cancer Center, Honolulu, HI (GM); Cancer Epidemiology Centre, Cancer Council Victoria, Melbourne, Victoria, Australia (GGG). 

The authors declare no conflicts of interest.

## Supplementary Material

Supplementary Data
