# Supplementary material for: Cirrus: An Automated Mammography-Based Measure of Breast Cancer Risk Based on Textural Features
Source: JNCI Cancer Spectr. 2018 Dec 7;2(4):pky057. doi: 10.1093/jncics/pky057 (PMC6649799; doi:10.1093/jncics/pky057)
Supplement: Supplementary Data [file pky057_supp.docx]

# Supplementary Materials

# Supplementary Methods

# Processing Film Mammograms

The pre-processing algorithm based on image processing techniques used to robustly segment the breast from the background noise, and to remove artefacts and labels, involves the following stages:

1. The edges of the image are blacked out to remove white edging strips.
2. Labels and textual markings are identified and removed using the Hough transform (Duda & Hart, 1972), which is designed to identify linear features (such as those that comprise letters and numbers), followed by image opening techniques (Haralick & Shapiro, 1992).
3. The intensity of the mammogram background is estimated from the mammogram, and areas with intensity lower than the background are blacked out. The background intensity is estimated by the following heuristic: find the histogram for all pixel intensity values less than 0.5, and use the most frequently occurring pixel value (i.e. the mode) in this range, multiplied by 1.25, as an estimate of the “average” background level. This exploits the fact that the background intensities are always below 0.5, and also accounts for the empirical observation that the range of background pixel intensities is roughly proportional to the mode of the background pixel intensities.
4. The breast itself is isolated using the Chan–Vese segmentation algorithm (Chan & Vese, 1999), which fits a flexible curve to an image with the aim of separating regions with different statistical properties.

Our algorithm takes a digitized film mammogram as an input and produces a new image that contains only the breast. For digital mammograms, which do not contain markings in the same manner as film mammograms, the first 2 steps will be unnecessary.

# GLCM Features

The GLCM class of features has a long history in the field of image processing. Essentially, they are derived as various functions of a special gray scale co-occurrence matrix. This matrix can be viewed as a table that counts the frequency of a particular gray scale level appearing in a pixel next to another gray scale level. Twenty features were extracted from this matrix (see Haralick, Shanmugam, & Dinstein, 1973; Soh & Tsatsoulis 1999; Clausi 2002 for more specific details). These features generally have clear physical interpretations, with many of them being analogs of quantities found in the physical sciences.

***Building the Co-occurrence Matrix***

The co-occurrence matrix is computed by first reducing (also known in the literature as *quantising*) the number of gray scale levels in the image from 4,096 to 8. Usually, this reduction is done by equally dividing the 4,096 gray levels into 8 bands (i.e. 0–511, 512–1023, etc.) and assigning each pixel in the image to the appropriate band. A problem with this approach is that it can be sensitive to differences in the digitizers themselves, as well as additional factors such the level of lighting used when digitizing the images. This can lead to inconsistencies; the same image, digitized on two different machines, could have quite different GLCM features.

To overcome this, we employed a modified approach to constructing the co-occurrence matrix. Essentially, instead of uniformly dividing the pixel gray scale levels into eight bands, we use the 0^th^, 12.5^th^, 25^th^, 37.5^th^, 50^th^, 62.5^th^, 75^th^, 87.5^th^, and 100^th^ percentiles to define the intervals that determine the eight new gray scale levels. Importantly, this simple modification to the algorithm renders the resulting GLCM features resistant to all image transformations that preserve the relative brightness of the pixels in the mammogram. For example, our GLCM features are robust to both changes in the level of lighting used to produce the mammogram, as well as to many of the standard image enhancement algorithms used by imaging and digitization software, such as histogram stretching and shaping.

The final GLCM matrix is an 8x8 table, called $P$, in which entry $P(i,j)$ is the probability that a pixel with a brightness level of *i* would be found next to a pixel with a brightness level of *j*. This is computed by counting the number of times a pixel with brightness level *i* is found next to a pixel with brightness level *j*, and then dividing each entry by the total number of pixels in the image. This table characterises how the brightness levels of the pixels vary within the image. For example, if the image consists of several large blocks of pixels with similar brightness, the diagonal entries of the co-occurrence matrix will be very large in comparison to the off-diagonal entries. In contrast, if the image was generated by randomly choosing a brightness level for each pixel, then almost every entry of the co-occurrence matrix would be the same.

***Computing the GLCM Features***

Given an occurrence matrix, $P,$ all GLCM features are defined as suitable functions of the entries of $P$. For example, the Energy, Entropy, Contrast and Homogeneity features are given by:

$$Energy=\sum_{i,j=1}^{8} {(P_{i,j})}^{2}$$

$$Entropy=\sum_{i,j=1}^{8} -P_{i,j}\log P_{i,j}$$

$$Contrast=\sum_{i,j=1}^{8} P_{i,j}\left( i-j \right)^{2}$$

$$Homogeneity=\sum_{i,j=1}^{8} \frac{P_{i,j}}{1+\left( i-j \right)^{2}}$$

These four features are excellent examples of the GLCM family of features as they demonstrate both how the GLCM features can capture intuitive and natural properties of an image, and also how many of the features can be highly correlated.

From the above formulae we can see that both the Energy and Entropy features will be larger if the image is composed in such a fashion that the likelihood of a pixel of brightness level *i* occurring next to a pixel with brightness level *j* is roughly equal for all pixel levels (i.e., the entries of the GLCM matrix $P$ are similar). This would occur if the image contains large areas in which the pixel brightness is randomly distributed. The Contrast feature will be larger if the GLCM matrix is such that the probability that pixel level *i* appears adjacent to pixel level *j* is high when level *i* is very different from level *j*. Two pixel levels being very different implies a large degree of contrast in the image, so it is clear that this feature implicitly captures the concept of “high contrast” in an image. On the other hand, the Homogeneity feature will be largest if the probability of pixel level *i* being adjacent to pixel level *j* is high if the two pixel levels are similar; high homogeneity therefore implies an image with large regions of similar pixel intensity.

It is obvious that the Homogeneity and Contrast features will be negatively correlated, as high Homogeneity implies low Contrast, and vice versa. The results of Table 3 confirm this intuition, as the adjusted odds-ratio for Contrast is 0.55 and the adjusted odds-ratio for Homogeneity is 1.75, which are roughly inverses of each other. A complete list of the GLCM textural features that we used in this paper can be found in Haralick, Shanmugam, & Dinstein, 1973; Soh & Tsatsoulis 1999; Clausi 2002.

# Computing the *Cirrus* Score

Once the 20 GLCM features have been calculated from the mammogram, the final *Cirrus* score is given by a linear combination of the features. The weightings of this linear combination were estimated using the Bayesian Lasso procedure, which helps reduce the effects of correlation between the predictors and is known to produce better predictive models than alternative procedures such as stepwise regression or the regular Lasso.

The GLCM features were all found to be unimodal, and the skewness and excess kurtosis for each measure, along with their corresponding standardised weights and posterior standard deviations are presented in Table 4. Ten of the features are approximately normally distributed, while 10 exhibit some level of skewness and evidence of heavier tails. We tried several non-linear transformations in combination with the Bayesian lasso but none gave an improved predictive performance. The features are each on different scales, so the weights are not directly comparable. The standardised weights are found by dividing the raw weight for each feature by their corresponding posterior standard deviation (roughly equivalent to the standard error) which allows for the relative contribution of each feature to be more readily assessed.

The *Cirrus* scores that were created by learning the optimal combination of GLCM features from each of the three different studies and were all highly correlated with each other. In particular, the score created using the Australian Cohort Study had a correlation of 0.85 with the score created using the Australian Case-Control Study, and a correlation of 0.92 with the score created using the Japanese Case-Control Study. The score created on the Australian Case-Control Study had a correlation of 0.82 with the score created on the Japanese Case-Control study. This supports the findings in Table 2.

The final *Cirrus* measure was found by combining each of the feature scores with the appropriate standardised weight from Table 4 multiplied by the corresponding posterior standard deviation. The skewness of the *Cirrus* measure for both cases and controls was approximately zero, and the excess kurtosis was approximately 0.2. The measure was therefore very close to normally distributed for both cases and controls. The mean difference between unadjusted *Cirrus* scores for cases and controls was 0.603 standard deviations of the controls. Using the formulae presented in Appendix D, the measure is expected to have an adjusted odds-ratio of around 1.82 (equivalent to an AUC of approximately 0.665). This is a reasonable match to the results for the final *Cirrus* measure tested on our data presented in Table 5, and to the empirical AUC of 0.662 (see Figure 3).

# Relationship between OR, AUC, IQQR and Δ as measures of risk discrimination

This appendix derives relationships between four different measures of risk discrimination ($\Delta, OR, AUC$ and $IQRR$; see below for definitions) for a continuous risk factor $X$ and a binary outcome $Y$, under a model where $X$ is normally distributed in both cases and controls. We prove the following formulae:

$$AUC=\Phi\left( \Delta/\sqrt{2} \right)$$

where

$$OR=\exp\left( \Delta\right),$$

$\Phi$ is the cumulative distribution function of the standard normal distribution, and

$$IQRR=\frac{\Phi\left( \Delta-b \right)}{\Phi\left( a-\Delta\right)},$$

where $a$ and $b$ can be solved for numerically, in general, but $a\approx\Phi^{-1}\left( 0.25 \right)=-0.6745$ and $b\approx\Phi^{-1}\left( 0.75 \right)=0.6745$ for a rare disease.

***The model***

Let $X$ and $Y$ be random variables representing a continuous risk factor and the affected status of a person randomly selected from a given population, respectively. We suppose that $Y=1$ corresponds to a case and $Y=0$ corresponds to a control, and we let $q_{1}=P\left( Y=1 \right)$ be the prevalence of disease, so that $q_{0}+q_{1}=1$ where $q_{0}=P\left( Y=0 \right)$. Throughout this appendix, we assume the distribution of the risk factor $X$ for cases is $N(\Delta,1)$ (i.e., the conditional distribution of $X$ given $Y=1$ is the univariate normal distribution with mean $\Delta$ and variance $1$) and the distribution of $X$ for controls is $N(0,1)$; see Wentzensen and Wacholder, 2013).

***Relationship between*** $\boldsymbol{AUC}$ ***and*** $\boldsymbol{\Delta}$

The area under the (receiver operating characteristic) curve, $AUC$, is the probability that a randomly selected case will have a higher value of the risk factor than a randomly selected control. Suppose we randomly (and uniformly) select a control and a case from the population, and let $X_{0}$ and $X_{1}$ be their risk factors, respectively. Then $X_{0}$ and $X_{1}$ are independent random variables, and under the above model they have marginal distributions $X_{0}\sim N(0,1)$ and $X_{1}\sim N(\Delta,1)$. Therefore $X_{1}-X_{0}\sim N(\Delta,2)$, so $Z=(X_{1}-X_{0}-\Delta)/\sqrt{2}$ is standard normal, hence

$$AUC=P\left( X_{1}>X_{0} \right)=P\left( \frac{X_{1}-X_{0}-\Delta}{\sqrt{2}}>-\frac{\Delta}{\sqrt{2}} \right)=P\left( Z>-\frac{\Delta}{\sqrt{2}} \right)=P\left( Z<\frac{\Delta}{\sqrt{2}} \right)= \Phi\left( \frac{\Delta}{\sqrt{2}} \right),$$

where $\Phi$ is the cumulative distribution function of the standard normal distribution.

***Relationship between*** $\boldsymbol{IQRR}$ ***and*** $\boldsymbol{\Delta}$

By definition,

$$IQRR=\frac{P\left( Y=1 | X>b \right)}{P\left( Y=1 | X<a \right)}$$

where $a$ and $b$ are the 25^th^ and 75^th^ percentiles of $X$ (respectively), so that they define the boundaries of the lower and upper quartiles. But we have

$$P\left( Y=1 | X<a \right)=\frac{P\left( X<a | Y=1 \right)P(Y=1)}{P(X<a)}=4q_{1}\Phi\left( a-\Delta\right),$$

since: $P\left( Y=1 \right)=q_{1}$ by definition; $P\left( X<a \right)=1/4$ because $a$ is the 25^th^ percentile of $X$; and the distribution of $X$ for cases is $N(\Delta,1)$. Similarly,

$$P\left( Y=1 | X>b \right)=4q_{1}\left( 1-\Phi\left( b-\Delta\right) \right)=4q_{1}\Phi\left( \Delta-b \right),$$

where the last step uses the fact that the standard normal distribution is symmetric. Taking the ratio of these two expressions gives

$$IQRR=\frac{\Phi\left( \Delta-b \right)}{\Phi\left( a-\Delta\right)}.$$

To determine $a$ and $b$, by the definition of $a$ we have

$$\frac{1}{4}=P\left( X<a \right)=\sum_{i=0}^{1} P\left( X<a | Y=i \right)P\left( Y=i \right)=q_{0}P\left( X<a | Y=0 \right)+q_{1}P\left( X-\Delta<a-\Delta| Y=1 \right)=q_{0}\Phi\left( a \right)+q_{1}\Phi\left( a-\Delta\right).$$

Similarly, we have

$$\frac{3}{4}=P\left( X<b \right)=q_{0}\Phi\left( b \right)+q_{1}\Phi\left( b-\Delta\right).$$

Given the disease prevalence $q_{1}$, these equations can be solved numerically for $a$ and $b$ (for any given $\Delta$) and these can be substituted into the equation above to give the $IQRR$ as a function of $\Delta$. Alternatively, for a rare disease, the distribution of $X$ is approximately the distribution of the risk factor in the controls, which is standard normal. Therefore $a\approx\Phi^{-1}\left( 0.25 \right)=-0.6745$ and $b\approx\Phi^{-1}\left( 0.75 \right)=0.6745$ for a rare disease.

***Relationship between*** $\boldsymbol{OR}$ ***and*** $\boldsymbol{\Delta}$

Define

$$\phi_{1}\left( x \right)= P\left( X=x | Y=1 \right)= \frac{1}{\sqrt{2\pi}}\exp\left( -\frac{(x-{\Delta)}^{2}}{2} \right)$$

and similarly

$$\phi_{0}\left( x \right)= P\left( X=x | Y=0 \right)= \frac{1}{\sqrt{2\pi}}\exp\left( -\frac{x^{2}}{2} \right) .$$

If $p_{1}\left( x \right)$ is the probability that a person with biomarker $X=x$ is a case, then

$$p_{1}\left( x \right)=P\left( Y=1 | X=x \right)= \frac{P\left( X=x \right|Y=1)P(Y=1)}{P(X=x)}=\frac{q_{1}\phi_{1}(x)}{q_{0}\phi_{0}\left( x \right)+ {q_{1}\phi}_{1}\left( x \right)},$$

since

$$P\left( X=x \right)= \sum_{y=0}^{1} P\left( X=x, Y=y \right)= \sum_{y=0}^{1} \left( X=x \right|Y=y) P\left( Y=y \right)=q_{0}\phi_{0}\left( x \right)+ {q_{1}\phi}_{1}\left( x \right).$$

Then the probability $p_{0}\left( x \right)$ that a person with biomarker $X=x$ is a control is ${1-p}_{1}\left( x \right)$, hence

$$p_{0}\left( x \right)=\frac{q_{0}\phi_{0}(x)}{q_{0}\phi_{0}\left( x \right)+ {q_{1}\phi}_{1}\left( x \right)}.$$

Therefore the odds, $odds\left( x \right)$, that a person with biomarker $X=x$ is a case is

$$odds\left( x \right)=\frac{p_{1}\left( x \right)}{p_{0}\left( x \right)}= \frac{q_{1}\phi_{1}(x)}{q_{0}\phi_{0}(x)}.$$

The odds ratio $OR\left( x \right)$ for a person with biomarker $X=x+1$ compared to a person with biomarker $X=x$ is

$$OR\left( x \right)=\frac{odds\left( x+1 \right)}{odds\left( x \right)}= \frac{\frac{q_{1}\phi_{1}(x+1)}{q_{0}\phi_{0}(x+1)}}{\frac{q_{1}\phi_{1}(x)}{q_{0}\phi_{0}(x)}}=\frac{\frac{\phi_{1}(x+1)}{\phi_{1}(x)}}{\frac{\phi_{0}(x+1)}{\phi_{0}(x)}} .$$

Using the formula for $\phi_{1}\left( x \right)$ above,

$$\frac{\phi_{1}\left( x+1 \right)}{\phi_{1}\left( x \right)}= \frac{\exp\left( {-\left( x- \Delta+1 \right)}^{2}/2 \right)}{\exp\left( {- \left( x- \Delta\right)}^{2}/2 \right)}=\exp\left( \Delta-x-\frac{1}{2} \right)$$

and similarly

$$\frac{\phi_{0}\left( x+1 \right)}{\phi_{0}\left( x \right)}= \exp\left( -x-\frac{1}{2} \right).$$

Substituting these equations into the expression for $OR\left( x \right)$ above gives

$$OR\left( x \right)=\frac{\exp\left( \Delta-x-\frac{1}{2} \right)}{\exp\left( -x-\frac{1}{2} \right)}= \exp\left( \Delta\right).$$

# References to Supplementary Materials

Chan, T. F., & Vese, L. A. (1999). An active contour model without edges. *Lect notes Comput Sci*, *1682*, 141–151.

Clausi, D. A. (2002). An analysis of co-occurrence texture statistics as a function of grey level quantization. *Canadian Journal of Remote Sensing, 28*(1), 45–62.

Duda, R. O., & Hart, P. E. (1972). Use of the Hough transformation to detect lines and curves in pictures. *Comm ACM, 15*(1), 11-15.

Haralick, R. M., Shanmugam, K., & Dinstein, I. (1973). Textural features of image classification. *IEEE Transactions on Systems, Man and Cybernetics, SMC-3*(6), 610–620.

Haralick, R. M., & Shapiro, L. G. (1992). *Computer and Robot Vision. Vol. 1*. Boston, MA: Addison-Wesley Longman Publishing Co.

Soh, L., & Tsatsoulis, C. (1999). Texture analysis of SAR sea ice imagery using gray level co-occurrence matrices. *IEEE Transactions on Geoscience and Remote Sensing, 37*(2), 780–795.

Wentzensen, N., & Wacholder, S. (2013). From differences in means between cases and controls to risk stratification: a business plan for biomarker development. *Cancer Discov*, *3*(2), 148–157. doi: 10.1158/2159-8290.CD-12-0196.

# Supplementary Table

**Supplementary Table 1. Characteristics of studies and of cases and controls within studies**

|  |  |  | **Caucasian**  **Cohort**  **(Australian)** | |  | **Caucasian**  **Case-Control**  **(Australian)** | |  | **Japanese**  **Cohort**  **(Japanese)** | |
| --- | --- | --- | --- | --- | --- | --- | --- | --- | --- | --- |
|  |  |  | Cases | Controls |  | Cases | Controls |  | Cases | Controls |
| **Studies** | |  |  |  |  |  |  |  |  |  |
| Sample size (no.) | |  | 590 | 1,695 |  | 354 | 944 |  | 292 | 292 |
| Digitiser/File extension/Conversion factors (%) | | | | | | |  |  |  |  |
|  | Kodak LS 85/iv2/0.000676 |  |  |  |  | 71 | 84 |  | 100 | 100 |
|  | Array 2905/dicom/0.000025 |  | 100 | 100 |  | 29 | 16 |  |  |  |
| **Subjects** | |  |  |  |  |  |  |  |  |  |
|  | Age (years) at mammogram |  | 58.5 | 58.3 |  | 47.5 | 48.2 |  | 57.0 | 60.2 |
|  | Body mass index (kg/m^2^) |  | 27.1 | 26.5 |  | 25.1 | 25.9 |  | 23.9 | 23.4 |
|  | Family history of breast cancer (%) ^a^ |  | 19.2 | 10.1 |  | 28.8 | 30.2 |  | 16.0 | 12.8 |
|  | Age (years) at menarche |  | 13.0 | 13.1 |  | 12.9 | 13.1 |  | 12.9 | 13.1 |
|  | Parous (%) |  | 84 | 87 |  | 73 | 82 |  | 85 | 89 |
|  | Age (years) at first birth |  | 25.3 | 25.2 |  | 25.3 | 25.2 |  | 25.6 | 25.4 |
|  | No. of children |  | 2.3 | 2.3 |  | 2.6 | 2.6 |  | 2.2 | 2.4 |
|  | Postmenopausal (%) |  | 67 | 67 |  | 51 | 60 |  | 70 | 78 |
|  | Any HRT use (%, postmenopausal) |  | 38 | 34 |  | 50 | 49 |  | 78 | 74 |

^a^ Percentage of first-degree relatives with breast cancer

# Supplementary Figures

**Supplementary Figure 1**. Mammograms from 12 women with high (top row) and low (bottom row) *Cirrus* risk measures. The high-risk mammograms are in the top 1% of *Cirrus* risk measures; the low risk mammograms are in the bottom 3% of *Cirrus* risk measures. All mammograms differ in conventional density risk percentiles from their corresponding *Cirrus* risk percentile by 40 percentage points or greater.

| High *Cirrus* Risk | 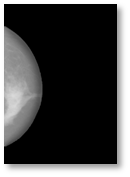 | 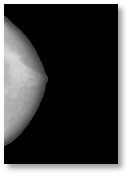 | 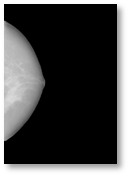 | 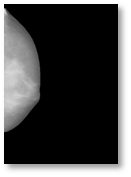 | 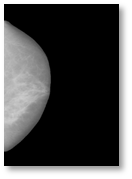 | 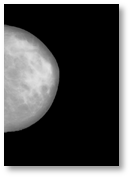 |
| --- | --- | --- | --- | --- | --- | --- |
| Low *Cirrus*  Risk | 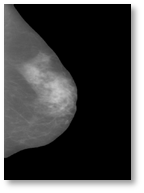 | 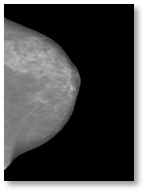 | 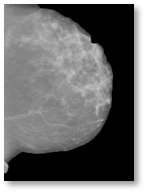 | 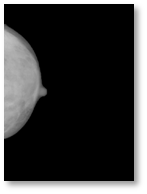 | 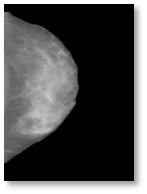 | 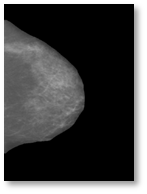 |

**Supplementary Figure 2**. Mammograms for the same low- and high-risk women as shown in Supplementary Figure 1, but processed (quantised) using the gray-scale co-occurrence matrix algorithm.

| High *Cirrus* Risk | 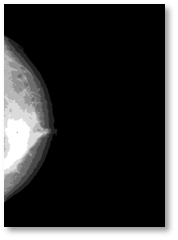 | 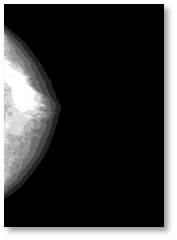 | 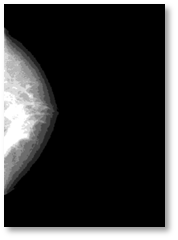 | 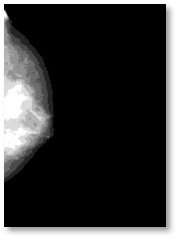 | 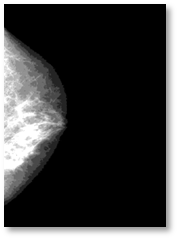 | 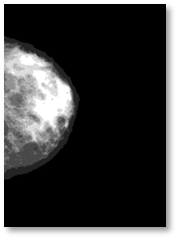 |
| --- | --- | --- | --- | --- | --- | --- |
| Low *Cirrus*  Risk | 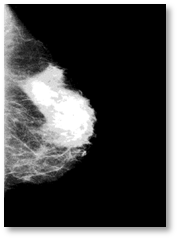 | 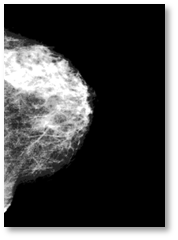 | 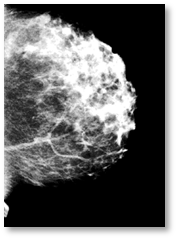 | 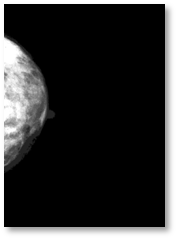 | 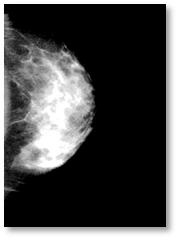 | 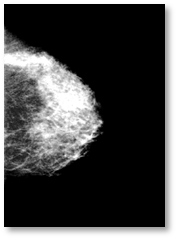 |
